# Downward Intergenerational Support and Well-Being in Older Chinese Adults

**DOI:** 10.3390/ijerph21111440

**Published:** 2024-10-30

**Authors:** Xin Liu, Shuying Bai

**Affiliations:** 1 Department of Sociology, Jimei University, Xiamen 361021, China; 2 Department of Sociology, Harbin Institute of Technology, Harbin 150001, China

**Keywords:** downward intergenerational support, self-esteem, older adults, gender

## Abstract

Life satisfaction, as an important indicator of subjective well-being, is crucial for older adults. This study aims to examine the effects of downward intergenerational financial and practical support on life satisfaction among older adults in China, while exploring the mediating role of self-esteem and the potential differences across gender. Data were collected from 507 older adults aged 60 and above in China. The findings indicate that both financial and practical support provided by older adults positively predict their life satisfaction. Mediation analysis reveals that self-esteem partially mediates the relationship between downward intergenerational support and life satisfaction. This suggests that helping their children enhances older adults’ sense of self-worth, which in turn improves their overall well-being. Gender differences were also observed: financial support had a stronger impact on life satisfaction for men, while practical support was more significant for women. These results highlight the influence of traditional gender roles and cultural expectations on the dynamics of intergenerational support in contemporary Chinese families.

## 1. Introduction

Intergenerational solidarity refers to the relationships and support between different generations, especially between parents and their children throughout the life course [1,2]. The concept of solidarity encompasses a range of diverse dimensions related to various aspects of life, such as affectional solidarity, functional solidarity, consensual solidarity, associational solidarity, structural solidarity, and normative solidarity. Intergenerational social support, as a concrete manifestation of intergenerational solidarity, particularly reflects functional solidarity. It refers to the emotional, financial, or practical assistance provided among family members. With the shrinking of the social network of the older people, family support, especially intergenerational support, is considered to be an important factor affecting the well-being and psychological health of the older people [3].

Although there has been less focus on the impact of providing support, relevant research indicates that helping others can improve emotional well-being in later life [4]. Downward intergenerational support, where older adults provide assistance to their children, has been found to positively influence the life satisfaction of older adults. This form of support can manifest in various ways, such as financial assistance, caregiving, and the transfer of knowledge and experience, and it plays a crucial role in maintaining the sense of purpose and connectedness among older adults. Downward intergenerational support is viewed as a means of reinforcing social ties, maintaining a sense of usefulness, and fulfilling generative roles within the family. Some scholars have found that older adults providing support to their children can improve their emotional state in later life [5]. Older adults who actively engage in supporting their children or grandchildren may also experience a heightened sense of purpose and identity, which can positively impact their overall life satisfaction [6].

Conversely, other researchers have argued that downward intergenerational support can lead to a decrease in life satisfaction, particularly when the support becomes burdensome [7]. This view is grounded in the notion that excessive demands placed on older adults—whether financial, emotional, or physical—can strain their resources and health, leading to stress, resentment, and decreased life satisfaction [8]. For example, older adults who are expected to provide extensive financial support to their adult children or who take on primary caregiving responsibilities for grandchildren may experience a significant reduction in their quality of life. The negative impact is particularly pronounced in situations where the support is perceived as obligatory or where the older adults feel they have little choice in the matter.

These contradictory findings indicate the need to further explore the underlying mechanisms of the relationships between them. Self-esteem is the emotional (or evaluative) component of the self-concept, reflecting how individuals perceive themselves and their overall sense of self-worth [9]. It can be conceptualized as a global evaluation of the self or as an evaluation of specific self-related domains. Self-esteem is shaped by various factors, including social roles, achievements, and the quality of interpersonal relationships. Family relationship is recognized as an important environmental factor influencing an individual’s self-esteem. The relationship between family functioning and self-esteem demonstrates cross-cultural consistency, with harmonious family relationships fostering the development of self-awareness [10].

In the context of intergenerational support, receiving too much support from children can reduce the self-efficacy of older adults and negatively impact their self-esteem [11,12]. However, providing financial, practical, or other forms of support to their adult children signifies that older adults still have the ability to contribute to the family. This can make it easier for them to find meaning in life and achieve a sense of self-worth [13], thereby boosting their self-esteem.

Some scholars have suggested that downward support may positively influence life satisfaction in older adults by enhancing their self-esteem, self-efficacy, or sense of competence [14]. Self-esteem, as a core component of psychological well-being, is believed to be influenced by the ability to provide support and maintain active participation within the family. However, this hypothesized pathway—whereby self-esteem mediates the relationship between downward intergenerational support and life satisfaction—has also not been empirically tested, leaving a significant gap in the literature.

Apart from the above, gender may also play a crucial role in shaping the psychological health of older adults and their experiences within intergenerational relationships. According to the life course and feminist perspectives, the gender-based division of practical in early economic and family spheres leads to different expectations, values, and sources of well-being in later life [15]. Research indicates that men and women differ in both the quantitative and qualitative aspects of social support, with these differences contributing to varying psychological outcomes in later life [16]. Women, who are often socialized into caregiving roles, derive greater self-worth and life satisfaction from providing support to younger generations. For older women, giving support—whether through financial assistance, emotional support, or caregiving—reinforces their identity as nurturers and significantly impacts their sense of self [17]. Building on this, the effects of intergenerational support on self-esteem and life satisfaction may vary by gender. It is hypothesized that the relationship between providing support to children and both self-esteem and life satisfaction is stronger for older women than for older men. For women, influenced by traditional gender roles and societal expectations, providing support is more closely aligned with their caregiving role, which may lead to greater self-esteem and life satisfaction.

### Research Aims

The aim of this study was to examine the effects of downward intergenerational financial support and practical support on older adults’ life satisfaction and self-esteem, assuming a positive effect, and to explore potential differences across gender.

## 2. Materials and Methods

### 2.1. Study Design and Participants

The data for this study were collected in Mengzhou City, Henan Province, China, a region renowned for its rich cultural heritage and historical significance. According to the local statistical yearbook of the year 2020, there were 79,005 individuals aged 60 and above in the area, comprising 22.54% of the total population. This proportion exceeds the national average of 18.7%. Among this older adult population, approximately 47.89% are male and 52.11% are female, highlighting the relevance of M City as a significant location for studying older adults.

The questionnaire used in this study was developed following the survey research framework proposed by Groves et al. [18]. The development process involved multiple stages to ensure the clarity and validity of the questions. First, a literature review was conducted to identify relevant constructs related to life satisfaction, self-esteem, and intergenerational support. Based on this review, initial draft items were generated. These items were then refined through cognitive interviews with a small group of older adults to assess comprehension and relevance. This was followed by a pretest to gather qualitative feedback on item wording and format. Only after these stages was the final version of the questionnaire created for data collection.

The sampling method employed in this study was convenience sampling. M City consists of 4 urban subdistricts (each containing approximately 2–8 communities), 6 towns (each with approximately 15–23 villages), and 1 township. Researchers established connections with community leaders from 2 communities and administrative heads from 3 villages. Participants in the study had to meet the following criteria: be at least 60 years old, be a local resident, and have children. Between June and August 2021, respondents were surveyed by trained interviewers accompanied by community workers.

The study included a sample of 507 individuals, with 234 males (46.2%) and 273 females (53.8%). Participants were aged 60 years and older, with the following age distribution: 60–65 years (35.7%), 66–69 years (16.0%), 70–75 years (38.1%), 76–79 years (7.7%), and 80 years or older (2.6%).

### 2.2. Measures

Life satisfaction (LS) was measured by one item: “All things considered, how satisfied are you with life”? Responses were recorded using a five-point Likert scale, where 1 represents “not at all satisfied” and 5 represents “completely satisfied”. This single-item measure has frequently been used in research as a reliable indicator of overall life satisfaction [19,20].

Downward intergenerational support were the focal independent variables. Downward intergenerational financial support (DIFS) was assessed by asking older adults how frequently they had provided financial assistance, food, or gifts to their children over the past year. Responses were recorded using a five-point Likert scale, where 1 = “never”, 2 = “seldom”, 3 = “sometimes”, 4 = “often”, and 5 = “very often”. Similarly, downward intergenerational practical support (DIPS) was measured by asking respondents how often they had assisted their children with household chores, care for grandchildren, or similar tasks over the past year, using the same five-point Likert scale.

We used self-esteem as the mediating variable. Self-esteem, as defined by Rosenberg, is a measure of self-worth and reflects an individual’s subjective evaluation of their own value as a person. The Rosenberg Self-Esteem Scale (SES) was adapted for the older adult population to better capture their subjective self-worth in a familial and community context [21]. The modified scale consists of five items: “I feel that I am a person of worth”, “I feel that I can still make important contributions to my family/community”, “I feel that I have many qualities to be proud of”, “I am satisfied with myself”, and “Overall, I tend to think of myself as a successful person”. These revisions ensure the scale more accurately reflects the self-esteem experiences of the older adult population.

Both the reliability and validity of the Self-esteems Scale are at good levels. We employed a structural equation model to conduct Confirmatory Factor Analysis (CFA) to assess the construct validity of multiple variables. The results indicated a good model fit (χ^2^ = 14.849, df = 5, *p* = 0.001). The fit indices also demonstrated strong alignment with the data: AGFI = 0.960, RMSEA = 0.062, CFI = 0.983, and TLI = 0.966. These outcomes suggest that the measurement model fits the data well. The Average Variance Extracted (AVE) exceeded 0.5, and the Composite Reliability (CR) was 0.842, surpassing the recommended threshold of 0.7, thereby providing robust support for both construct validity and convergent validity. The Kaiser–Meyer–Olkin (KMO) value and Bartlett’s test of sphericity were acceptable (KMO = 0.800, Bartlett’s test significance = 0.000), confirming the suitability for factor analysis. Internal consistency was verified through Cronbach’s alpha, which was 0.820—well above the 0.7 benchmark—indicating high reliability.

Gender (1 = male; 0 = female) was used as the important moderator. In addition, we surveyed the respondents’ age, education, and activities of daily living (ADLs) to minimize the influence of other exogenous variables. Age was categorized into five groups: 60–65, 66–69, 70–75, 76–79, and 80 years and above. Education was classified into five levels: primary school or less, middle school, high school, associate degree, and bachelor’s degree or higher. ADLs were measured on a scale ranging from 1 to 5, with 1 indicating complete dependence and 5 indicating complete independence.

### 2.3. Data Analysis

In accordance with Baron and Kenny’s criteria for mediation, three conditions were tested: first, the relationship between the independent variable and the dependent variable was examined; second, the relationship between the independent variable and the mediator was assessed; and third, the total effect of the independent variable on the dependent variable, accounting for the mediator, was evaluated [22]. Full mediation is indicated if the significant relationship between the independent variable and the dependent variable becomes non-significant when controlling for the mediator. Partial mediation is indicated if the relationship is weakened but remains significant [23]. Based on this method, a multivariable linear regression analysis was conducted to explore the relationship between downward intergenerational support and life satisfaction using SPSS 26.0 IBM Corp., Armonk, NY, USA) at first. The independent variables, downward intergenerational support (DIFS and DIPS), was entered first, followed by control variables including age, education, and ADLs. Next, mediation analysis was performed using SPSS 26.0 to examine the mediating effect of self-esteem in the relationship between downward intergenerational support and life satisfaction. To explore whether there are differences in the effects of downward intergenerational support on self-esteem and life satisfaction between older men and women, we also conducted heterogeneity tests. The results are summarized separately for each gender group as well as for the entire sample.

To enhance the robustness of parameter estimates and address potential limitations of traditional inferential statistics, we employed a non-parametric percentile bootstrap test. This method provides more reliable confidence intervals for mediation effects, which is particularly useful given the non-normality of the data. The method is realized by the process extension macro of SPSS software.

## 3. Results

### 3.1. Descriptive Statistics

Table 1 shows the sampling distribution of older adults aged 60 and above. The total population in the area of adults aged 60 and above is 79,005, while the sample includes 507 participants. In the population, 47.89% are males and 52.11% are females, which is similar to the sample distribution of 46.15% males and 53.85% females. Most of the population (58.94%) is aged 60–69, followed by 29.18% aged 70–79 and 11.88% aged 80 and above. In the sample, 51.68% are aged 60–69, 45.76% are aged 70–79, and 2.56% are aged 80 and older. Overall, the sample closely matches the demographic profile of the local population of older adults, indicating that the sample is representative.

Table 2 presents the descriptive statistics of the variables. The mean score for Downward Intergenerational Financial Support (DIFS) was 2.52 out of 5, and for Downward Intergenerational Practical Support (DIPS), it was 3.20 out of 5. SES had a mean of 3.40 out of 5, indicating generally high levels among participants. Life satisfaction scored a mean of 3.51 out of 5, reflecting a relatively high level of satisfaction. Gender distribution was nearly equal with a mean of 0.54. The mean age was 2.25, with education at 1.76, and Activities of Daily Living (ADLs) averaged 4.17 out of 5, suggesting participants were largely independent in their daily activities.

Table 3 shows the correlation coefficient results among the variables. Downward Intergenerational Financial Support is positively correlated with SES (r = 0.39, *p* < 0.001) and Life Satisfaction (r = 0.32, *p* < 0.001). Downward Intergenerational Practical support is positively correlated with SES (r = 0.44, *p* < 0.001) and Life Satisfaction (r = 0.34, *p* < 0.001). SES is positively correlated with Life Satisfaction (r = 0.40, *p* < 0.001). These results provide initial support for the hypothesized relationships among the variables.

### 3.2. OLS Regression Results

Multiple linear regression equations were used to test the main effects and intermediary effects of each independent variable, as well as the moderating effects of gender, in Table 4 and Table 5.

Table 4 shows the relationship between DIFS, SES and life satisfaction, as well as the moderating role of gender in these relationships. The findings show that DIFS significantly and positively predicts older adults’ life satisfaction (Model 3: β = 0.30, *p* < 0.001). Additionally, DIFS is a significant positive predictor of older adults’ self-esteem (Model 7: β = 0.35, *p* < 0.001). Models 3 and Model 4 demonstrate that when SES is included in the model, the effect coefficient of DIFS on life satisfaction decreases from 0.30 to 0.19. This reduction indicates that SES partially mediates the relationship between DIFS and life satisfaction among older adults.

Table 4 also examines the role of gender in the relationship between DIFS and life satisfaction, as well as between DIFS and SES. In Model 1 and Model 2, DIFS was positively associated with life satisfaction for both men (β = 0.31, *p* < 0.001) and women (β = 0.29, *p* < 0.001), with a slightly stronger effect for men. Similarly, in Model 5 and Model 6, DIFS was positively related to SES for both genders, with men (β = 0.44, *p* < 0.001) again showing a slightly stronger effect than women (β = 0.22, *p* < 0.01). These findings suggest that while DIFS positively influences both life satisfaction and SES across genders, the effect is somewhat stronger for men.

Table 5 shows the relationship between DIPS, SES, and life satisfaction, as well as the moderating role of gender in these relationships. The findings show that DIPS significantly and positively predicts older adults’ life satisfaction (Model 3: β = 0.29, *p* < 0.001). Additionally, DIPS is a significant positive predictor of older adults’ self-esteem (Model 7: β = 0.42, *p* < 0.001). Models 3 and Model 4 demonstrate that when SES is included in the model, the effect coefficient of DIPS on life satisfaction decreases from 0.29 to 0.17. This reduction indicates that SES partially mediates the relationship between DIPS and life satisfaction among older adults.

Table 5 also examines the role of gender in the relationship between DIPS and life satisfaction, as well as between DIPS and SES. In both Model 1 and Model 2, DIPS is positively associated with life satisfaction for both men (β = 0.27, *p* < 0.001) and women (β = 0.33, *p* < 0.001), with the effect being slightly stronger for women. However, no significant difference is observed in the effect of DIPS on SES between men and women. These findings suggest that while DIPS positively influences life satisfaction across genders, the effect is somewhat more pronounced for women.

### 3.3. Non-Parametric Percentile Bootstrap Test Results

On the basis of regression analysis, we used the non-parametric percentile bootstrap test to test the main effects and intermediary effects of each independent variable. The 95% confidence interval can be used to assess whether the intermediary path is established or not. If the confidence interval of the effect does not include 0, the effect is significant, indicating that the intermediary path is established.

As Table 6 shows, DIFS affects life satisfaction through SES, with a total indirect effect of 0.11, accounting for 39.3% of the total effect in the model (0.11 out of 0.28). This indicates that SES explains 39.3% of the mediating effect of DIFS on life satisfaction. The direct effect of DIFS on life satisfaction is 0.17, which accounts for the remaining 60.7% of the total effect. Similarly, DIPS affects life satisfaction through SES, with a total indirect effect of 0.08, accounting for 40.0% of the total effect in the model (0.08 out of 0.20). This suggests that SES explains 40.0% of the mediating effect of DIPS on life satisfaction. The direct effect of DIPS on life satisfaction is 0.12, accounting for the remaining 60.0% of the total effect.

## 4. Discussion

In this study, we aimed to explore the mediating role of self-esteem in the relationship between downward intergenerational financial support and older adults’ life satisfaction, while also exploring potential differences across gender. The results revealed that self-esteem played a mediating role in the association between downward intergenerational financial support and older adults’ life satisfaction. The impact of downward intergenerational financial support on the life satisfaction of older adults is understood through a direct and indirect path (through self-esteem). The current research found that downward intergenerational financial support was positively associated with the life satisfaction of older adults. This result is in agreement with the findings of an earlier study, which showed that older adults who provide support to their adult children often report greater happiness [24]. Self-esteem Enhancement Theory asserts that individuals are inclined to engage in activities that boost their self-esteem, which in turn promotes their overall psychological well-being [25]. This theory suggests that even when individuals feel they are giving more support than they receive, the act of providing support can still enrich their personal and social resources. Altruistic behaviors, or those performed without expecting immediate or specific rewards, can be particularly fulfilling and restorative [26]. Therefore, in intimate relationships, providing support to close individuals can also positively impact the well-being of the support provider. For older adults, providing support can enhance overall well-being [27]. Downward intergenerational financial support enhances older adults’ self-esteem by reinforcing their sense of purpose and value within the family. When they provide financial assistance to their children, they often feel more connected and appreciated, which boosts their self-worth, as the act of giving itself serves as a source of emotional fulfillment [28]. However, the role of gender in the above relationship did not align with our initial expectations. Although both men and women experienced increased self-esteem from providing financial support, contrary to our hypothesis that women would benefit more, the results indicated that this effect was slightly stronger for men. This discrepancy can be explained by the traditional gender division of labor in Chinese families. In China, the division of labor within the family remains relatively traditional, with women generally expected to handle housework and caregiving, while men are viewed as the primary breadwinners [29]. For older men, providing financial support to their children reinforces their status as the heads of the family, which in turn enhances their self-satisfaction and overall life satisfaction.

The results also showed that self-esteem mediated the association between downward intergenerational practical support and life satisfaction of older adults. These contributions reinforce their roles as caregivers or problem-solvers, which bolsters their self-esteem. By helping with tasks such as cooking, cleaning, or taking care of grandchildren, older adults demonstrate their ongoing utility and importance within the family structure. The feeling of being needed and the ability to contribute to the family’s daily functioning provides emotional satisfaction and strengthens their self-worth. This sense of competence and involvement nurtures self-esteem, which in turn enhances their life satisfaction. Previous studies have also found that when children provide excessive support to their parents, it can undermine their desire for independence and lead to feelings of guilt, inadequacy, and other negative emotions [30]. Conversely, older adults who actively engage in supporting their children or grandchildren may experience a heightened sense of purpose and identity, positively influencing their overall life satisfaction [6]. This positive correlation is often observed when the support provided by older adults is voluntary, manageable, and aligned with their abilities and resources. Our study also found a stronger relationship between downward intergenerational practical support and self-esteem in older women than in older men, suggesting that women may derive greater psychological benefits from providing practical support to their children or grandchildren. This difference could be attributed to traditional gender roles that encourage women to be nurturers, thus enhancing their sense of self-worth through caregiving activities and other forms of practical support. A recent study conducted in China, Japan, South Korea, and Taiwan showed that women still bear more household responsibilities than men [31]. In China, older women spend more time caring for their grandchildren compared to older men. This differentiation in roles may explain why downward intergenerational practical support has a more significant impact on women’s self-esteem, as it aligns with their culturally defined responsibilities and the fulfillment of these roles enhances their self-esteem and well-being.

Xiaotong Fei, a renowned Chinese sociologist, introduced the concept of the “differentiated mode of association”, which emphasizes the family-centered relational network in Chinese society [32]. According to this theory, older adults in China are inclined to make sacrifices for their children because their self-awareness and actions are oriented around family interests and the continuity of the family line. In this cultural framework, individual self-worth is often realized through maintaining and reinforcing these family connections. In traditional Chinese culture, the family is the core of society, and older adults’ dedication to their family reflects their personal value. Although China is undergoing rapid social and economic changes, which are reshaping traditional family structures and roles, downward intergenerational support remains prevalent [33]. The finding that older adults still derive significant well-being from providing such support suggests that, despite modern shifts, contributing to the family (especially to their children) remains a critical source of identity and satisfaction for the older adults—at least in the areas we surveyed.

Our analyses also reveal significant findings regarding the impact of age, education, and Activities of Daily Living (ADLs) on life satisfaction among older adults. Age demonstrates a negative association with life satisfaction, indicating that as individuals grow older, they tend to report lower levels of satisfaction, likely due to challenges associated with aging such as health decline and loss of social roles. In contrast, ADLs consistently show a positive relationship with life satisfaction, suggesting that greater independence and the ability to perform daily activities are crucial for enhancing overall well-being. Education presents a more nuanced effect; while it positively influences life satisfaction for women in the context of downward intergenerational practical support, it does not exhibit a significant impact for men. This indicates that educational attainment may enhance personal fulfillment differently across genders. Overall, these findings highlight the need to address various factors contributing to life satisfaction in older adults, particularly by promoting independence in daily living and considering the different effects of education. Future research could explore longitudinal studies to assess how changes in these factors over time affect life satisfaction. Additionally, investigating the role of cultural and contextual influences on the relationship between education, ADLs, and life satisfaction may provide deeper insights into optimizing well-being among diverse populations of older adults.

It is important to acknowledge that our study has several limitations. First, regarding sample selection, we utilized a convenience sampling method, which inherently limits the representativeness of our findings. This approach may introduce bias, as the sample may not accurately reflect the broader population of older adults in China, making it difficult to generalize our findings to older adults from different geographic areas or socio-economic backgrounds. Second, we focused solely on unidirectional downward intergenerational support, without considering the reciprocal nature of support exchanges. Future research should explore how balanced intergenerational support impacts the psychological well-being of older adults. Finally, the significance of these results obtained from a single ethnic group raises questions about their applicability to the global population. Cultural differences in familial roles, support systems, and societal norms may influence how intergenerational support affects the well-being of older adults in other contexts. Therefore, while our findings provide valuable insights, further research is needed to investigate these dynamics across diverse ethnic groups and cultural settings to enhance their relevance and applicability worldwide.

## 5. Conclusions

In conclusion, this study highlights the critical mediating role of self-esteem in the relationship between downward intergenerational financial and practical support and the life satisfaction of older adults. The findings reveal that both forms of support significantly enhance older adults’ self-esteem, which subsequently improves their overall life satisfaction. We also explored potential differences across gender. While both men and women experienced increased self-esteem from providing financial support, contrary to our hypothesis that women would benefit more, the results indicated that this effect was slightly stronger for men. Conversely, a stronger relationship was observed between downward intergenerational practical support and self-esteem in older women than in older men, suggesting that women may derive greater psychological benefits from engaging in caregiving activities. This nuanced understanding of gender roles emphasizes the importance of intergenerational support dynamics in fostering the well-being of older adults. Future research should further investigate the nuanced effects of different types of intergenerational support on the well-being of older adults, as well as the broader implications for mental health and social engagement in later life across various cultural contexts.

## Figures and Tables

**Table 1:** Sampling distribution.

Characteristic		Population over 60 Years Old	Sample Size
		(*n* = 79,005)	(*n* = 507)
		*n* (%)	*n* (%)
Gender	Male	37,835 (47.89%)	234 (46.15%)
	Female	41,170 (52.11%)	273 (53.85%)
Age	60–69 years old	46,565 (58.94%)	262 (51.68%)
	70–79 years old	23,055 (29.18%)	232 (45.76%
	80 and above	9385 (11.88%)	13 (2.56%)

**Table 2:** Descriptive statistics of variables.

Variables	Mean	SD
DIFS	2.52	0.82
DIPS	3.20	1.21
SES	3.40	0.52
Life satisfaction	3.51	0.72
Gender	0.54	0.50
Age	2.25	1.10
Education	1.76	0.86
ADLs	4.17	1.09

**Table 3:** The correlation matrix between the variables.

Variables	1	2	3	4	5	6	7	8
1. DIFS	1	0.09 *	0.39 ***	0.32 ***	−0.07	−0.06	0.35 ***	0.14 **
2. DIPS	0.09 *	1	0.44 ***	0.34 ***	−0.01	−0.39 ***	0.20 ***	0.32 ***
3. SES	0.39 ***	0.44 ***	1	0.40 ***	0.12 **	−0.17 ***	0.24 ***	0.22 ***
4. Life satisfaction	0.32 ***	0.34 ***	0.40 ***	1	−0.04	−0.16 ***	0.14 **	0.28 ***
5. Gender	−0.07	−0.01	0.12 **	−0.04	1	0.12 ***	−0.20 ***	−0.12 **
6. Age	−0.06	−0.39 ***	−0.17 ***	−0.16 ***	0.12 ***	1	−0.37 ***	−0.48 ***
7. Education	0.35 ***	0.20 ***	0.24 ***	0.14 **	−0.20 ***	−0.37 ***	1	0.28 ***
8. ADLs	0.14 **	0.32 ***	0.22 ***	0.28 ***	−0.12 **	−0.48 ***	0.28 ***	1

Note: ***, *p* < 0.001; **, *p* < 0.01; *, *p* < 0.05.

**Table 4:** Hierarchical regression analysis for DIFS, SES, gender, and life satisfaction.

Variable	Life Satisfaction				SES		
	Model 1	Model 2	Model 3	Model 4	Model 5	Model 6	Model 7
	Males (*n* = 234)	Females (*n* = 273)	Total (*n* = 507)	Total (*n* = 507)	Males (*n* = 234)	Females (*n* = 273)	Total (*n* = 507)
DIFS	0.31 ***	0.29 ***	0.30 ***	0.19 ***	0.44 **	0.22 ***	0.35 ***
SES	/	/	/	0.29 ***	/	/	/
Age	−0.13	−0.02	−0.05	−0.03	−0.16 *	−0.01	−0.08
Education	−0.09	−0.14 *	−0.04	−0.06	0.12 *	0.08	0.06
ADLs	0.18 **	0.24 ***	0.22 ***	0.19 ***	0.08	0.16 *	0.11 *
Adjusted R^2^	0.22	0.12	0.15	0.22	0.31	0.09	0.18
F	17.44 ***	10.15 ***	23.30 ***	29.44 ***	27.58 ***	7.96 ***	28.44 ***

Note: ***, *p* < 0.001; **, *p* < 0.01; *, *p* < 0.05.

**Table 5:** Hierarchical regression analysis for gender, DIPS, SES, and life satisfaction.

Variable	Life Satisfaction				SES		
	Model 1	Model 2	Model 3	Model 4	Model 5	Model 6	Model 7
	Males (*n* = 268)	Females (*n* = 239)	Total (*n* = 507)	Total (*n* = 507)	Males (*n* = 268)	Females (*n* = 239)	Total (*n* = 507)
DIPS	0.27 ***	0.33 ***	0.29 ***	0.17 ***	0.42 ***	0.42 ***	0.42 ***
SES	/	/	/	0.29 ***	/	/	/
Age	0.10	0.02	0.07	0.04	0.03	0.14 *	0.09
Education	−0.03	0.15 *	0.05	0.01	0.21 ***	0.16 **	0.16 ***
ADLs	0.20 **	0.20 **	0.20 ***	0.18 **	0.12	0.08	0.08
Adjusted R^2^	0.11	0.22	0.15	0.21	0.29	0.20	0.22
F	8.94 ***	17.73 ***	22.37 ***	27.93 ***	17.55 ***	24.59 ***	36.73 ***

Note: ***, *p* < 0.001; **, *p* < 0.01; *, *p* < 0.05.

**Table 6:** Bootstrap analysis of the relationships among DIFS, DIPS, SES, and life satisfaction.

Path	Total Effect		Direct Effect		Indirect Effect	
	Effect Value	95% Confidence Interval	Effect Value	95% Confidence Interval	Effect Value	95% Confidence Interval
DIFS → life satisfaction	0.28	(0.21, 0.35)	0.17	(0.09, 0.24)		
DIFS → SES → life satisfaction					0.11	(0.07, 0.15)
DIPS → life satisfaction	0.20	(0.15, 0.25)	0.12	(0.07, 0.17)		
DIPS → SES → life satisfaction					0.08	(0.05, 0.11)

## Data Availability

Data are unavailable due to privacy and ethical restrictions.
